# Cell-Laden Gelatin Methacryloyl Bioink for the Fabrication of Z-Stacked Hydrogel Scaffolds for Tissue Engineering

**DOI:** 10.3390/polym12123027

**Published:** 2020-12-17

**Authors:** Jeong Wook Seo, Joon Ho Moon, Goo Jang, Woo Kyung Jung, Yong Ho Park, Kun Taek Park, Su Ryon Shin, Yu-Shik Hwang, Hojae Bae

**Affiliations:** 1 Department of Stem Cell and Regenerative Biotechnology, KU Convergence Science and Technology Institute, Konkuk University, Seoul 05029, Korea; wjddnr9302@naver.com; 2 LARTBio Inc., Seoul 06221, Korea; joonhomoon@lartbio.com; 3 Laboratory of Theriogenology and Biotechnology, Department of Veterinary Clinical Science, College of Veterinary Medicine and the Research Institute of Veterinary Science, Seoul National University, 1 Gwanak-ro, Gwanak-gu, Seoul 08826, Korea; snujang@snu.ac.kr; 4 Department of Microbiology, College of Veterinary Medicine and Research Institute for Veterinary Science, Seoul National University, 1 Gwanak-ro, Gwanak-gu, Seoul 08826, Korea; fanta2@snu.ac.kr (W.K.J.); yhp@snu.ac.kr (Y.H.P.); 5 Department of Biotechnology, Inje University, 197 Injero, Gimhae-si 50834, Gyeongsangnam-do, Korea; ktpark@inje.ac.kr; 6 Division of Engineering in Medicine, Department of Medicine, Harvard Medical School, Brigham and Women’s Hospital, Cambridge, MA 02139, USA; sshin4@bwh.harvard.edu; 7 Department of Maxillofacial Biomedical Engineering and Institute of Oral Biology, School of Dentistry, Kyung Hee University, Seoul 02447, Korea; yshwang@khu.ac.kr

**Keywords:** polymerization, Z-stacking bioprinting, Z-stacked scaffold, GelMA, tissue engineering

## Abstract

Hydrogel-based scaffolds have been widely used to fabricate artificial tissues capable of replacing tissues and organs. However, several challenges inherent in fabricating tissues of large size and complex morphology using such scaffolds while ensuring cell viability remain. To address this problem, we synthesized gelatin methacryloyl (GelMA) based bioink with cells for fabricating a scaffold with superior characteristics. The bioink was grafted onto a Z-stacking bioprinter that maintained the cells at physiological temperature during the printing process, without exerting any physical pressure on the cells. Various parameters, such as the bioink composition and light exposure time, were optimized. The printing accuracy of the scaffolds was evaluated using photorheological studies. The internal morphology of the scaffolds at different time points was analyzed using electron microscopy. The Z-stacked scaffolds were fabricated using high-speed printing, with the conditions optimized to achieve high model reproducibility. Stable adhesion and high proliferation of cells encapsulated within the scaffold were confirmed. We introduced various strategies to improve the accuracy and reproducibility of Z-stack GelMA bioprinting while ensuring that the scaffolds facilitated cell adhesion, encapsulation, and proliferation. Our results demonstrate the potential of the present method for various applications in tissue engineering.

## 1. Introduction

The purpose of tissue engineering is to develop artificial tissues using functional biomaterials that can replace damaged tissues and organs [1,2,3]. Recently, hydrogel-based scaffolds have attracted attention because they provide an aqueous environment, mimic the extracellular matrix (ECM), and exhibit excellent nutrient permeability [4,5,6]. However, methods for the rapid fabrication of sophisticated tissues with guaranteed cell viability in actual size are a major challenge. Recently, many studies have reported the fabrication of complex hydrogel-based scaffolds using 3D printers with bioinks composed of a mixture of biomaterials and cells [7,8]. In a typical extrusion-type 3D bioprinting method, a bioink that maintains structural integrity is extruded based on a three-dimensional (3D) axis. This approach is widely used because it allows intuitive movement and permits simple user adjustment [9,10]. 

Gelatin methacryloyl (GelMA) has gained considerable attention as a core material for tissue engineering owing to its excellent biocompatibility and tunable physicochemical properties [11,12,13]. In addition, compared to other biocompatible hydrogels, GelMA has properties similar to the extracellular matrix, making it compatible with a variety of cells [13,14]. However, GelMA has some limitations in 3D printing. First, the gelatin molecular chain is much longer than the photo-crosslinking group, and it is difficult to optimize the degree of reaction with a complex mixture with various functional groups [15]. Second, below the physiologically active temperature, helix-coil transition occurs, making the normal printing process difficult [16]. Third, pure GelMA scaffold is considered a material with weak mechanical properties. Due to the influence of gravity, pure GelMA has limitations even at low stacking heights. In the case of extrusion printing, an approach is taken to give up some biocompatibility and add reinforcing agents such as nanocellulose [17]. Therefore, printing pure GelMA without mixing other polymers is an important task while maintaining excellent biocompatibility [18].

In this study, we adopted the Z-stacking printing method in which GelMA is stacked in the reverse direction of gravity on an inverted plate to make a scaffold with high cell affinity without mixing with other polymers. This method made it possible to fabricate a large-scale scaffold (59.41 mm (w) × 33.45 mm (l) × 8.54 mm (h)) despite the weak mechanical strength of GelMA. To do this, the Z-stacking 3D printer was customized to maintain a physiologically active temperature environment so that GelMA does not coil at low temperatures. To optimize the degree of photoreaction of GelMA, the light emission wavelength band of the printer and the reaction wavelength band of GelMA were synchronized using vitamin K_1_. In addition, the light exposure time was optimized through photo-rheological analysis. The Z-stacked GelMA scaffold fabricated under such optimized conditions has high quality that can form a 550-micrometer channel while maintaining excellent biocompatibility. As a result, it was possible to completely reproduce the complex structure such as human ear in actual size and confirm the high viability of the embedded cells.

## 2. Materials and Methods

### 2.1. Materials

Gelatin (Type A, 300 bloom from porcine skin), methacrylic anhydride (MA), lithium phenyl-2,4,6-trimethylbenzoylphosphinate (≥95%, LAP), and vitamin K_1_ (viscous liquid) were purchased from Sigma-Aldrich (St. Louis, MO, USA). Fetal bovine serum (FBS), penicillin streptomycin (p/s), high glucose Dulbecco’s modified Eagle’s medium (DMEM), Dulbecco’s phosphate buffered saline (DPBS), phosphate buffered saline (PBS, pH 7.4), and 0.05% trypsin-EDTA solutions were purchased from WelGene (Daegu, Gyeongbuk, Korea). All other chemical agents used in this study were of analytical grade.

### 2.2. Cell Culture

Bovine ear fibroblast cells (BEFCs), a generous gift from LARTbio, Seoul were cultured in high glucose DMEM supplemented with 10% FBS and 1% p/s. The cells were incubated at 37 °C in 5% CO_2_. Cells were passaged using 0.05% trypsin-EDTA approximately twice a week, and the medium was exchanged every 2 d.

### 2.3. Gelatin Methacrylate Synthesis

**Figure 1:**
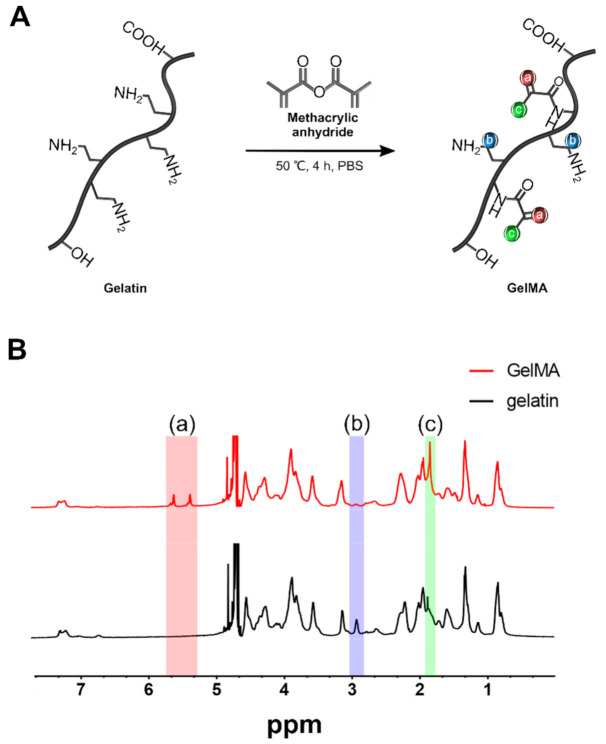
(**A**) GelMA was synthesized by reacting gelatin with methacrylic anhydride (MA) to introduce a methacryloyl substituent to the reactive group of the lysine residue. The methylene group of lysine in GelMA is indicated by blue (b), and the new functional groups are indicated by red (a) and green (c). (**B**) ^1^H NMR of gelatin and GelMA.

GelMA was synthesized as described previously [19] (Figure 1A). Briefly, 10% (*w/v*) type A porcine skin gelatin was added to PBS and stirred at 50 °C until fully dissolved. Methacrylic anhydride (MA) was then added at a rate of 1 mL/min until a concentration of 15% (*v*/*v*) was achieved. The solution was allowed to react for 4 h at 50 °C. The reaction was stopped by adding 400% (*v*/*v*) PBS at 40 °C. Then, the mixture was dialyzed against distilled water for 1 week using 12~14 kDa cutoff dialysis tubing to remove residual methacrylic anhydride. Finally, the solution was lyophilized for 4 d to obtain fully dried pure GelMA.

### 2.4. Preparation and Fabrication of Bioink

For the preparation of bioink, lyophilized GelMA was completely dissolved (10% [*w*/*v*]) in DPBS at 37 °C with 2% (*v*/*v*) FBS, 1% p/s, 0.5% (*w*/*v*) LAP, and 0.1% (*w*/*v*) vitamin K_1_. Cultured BEFCs were detached from the culture plate and added to the GelMA solution at a concentration of $2\times 10^{6}$ cells/mL. The prepared bioink was then transferred to an IM2 (Z-stacking 3D printer; Carima, Seoul, Korea) resin tank that was preheated to 37 °C. The thickness of the single layer was set at 100 μm for printing Z-stacks. The initial three layers were exposed to light for a long period (10 s) to prevent cross-linked 3D constructs from falling off the plate. The light exposure after the initial layer was set to 6 s. After the printing process, the scaffold was removed from the plate and washed twice with DPBS at 37 °C. Finally, the scaffold was cultured in high glucose DMEM supplemented with 10% FBS and 1% p/s.

### 2.5. ^1^H nuclear Magnetic Resonance (^1^H NMR) Spectroscopy

^1^H NMR spectroscopy was performed to determine the degree of substitution (DS) of GelMA. Gelatin and GelMA were dissolved in D_2_O and analyzed using a 500 MHz FT-NMR spectrometer (Varian; Palo Alto, CA, USA).

### 2.6. Photorheological Analysis

Photorheology was performed using a HAKKE MARS 40 (Thermo Fisher Scientific, Waltham, MA, USA) equipped with a UV module accessory. To investigate the rheological behavior of GelMA when the Z-stacking printer was irradiated with light, a light source with a wavelength similar to that of IM2 was produced by an OmniCure LX 505 (Lumen Dynamics; Mississauga, ON, Canada) equipped with a 400 nm LED channel. Irradiation was guided through the collimator and reflected towards the parallel glass plate geometry. The LED intensity was measured from the glass plate geometry and set to the same intensity as IM2. About 10 μL of cell-free bioink was loaded between the plates, and the spacing was set to 100 μm. The oscillation experiment was conducted at 37 °C, with an oscillating shear strain of 0.01% and frequency of 1 Hz. Rheological measurements and the UV irradiation trigger were operated simultaneously without any delay.

### 2.7. Printing Accuracy

To ensure reproducibility of the printed scaffold models, the printing accuracy was evaluated using a hollow tube design. Hollow tube scaffold (27.2 mm (w) × 11 mm (l) × 2.5 mm (h)) with one inlet extending upward and 14 tubes with different diameters (250~1000 μm) connected to the inlet was used. Based on the information obtained through the photorheological analysis, printing was performed under three conditions: LED exposure times of 5, 6, and 7 s. To accurately examine the hollow tube formation of the printed scaffold, 0.065% (*w*/*v*) eosin Y solution was perfused into the inlet through a syringe. Then, the fluorescence emission from the hollow tube was observed under UV illumination using OmniCure S2000 (Lumen Dynamics; Mississauga, ON, Canada) with a 450–550 nm external filter.

### 2.8. Scanning Electron Microscopy (SEM)

To confirm the internal morphological features of cell-free Z-stacked GelMA scaffolds over the course of time—1, 3, and 5 d—a rectangular parallelepiped scaffold (15 mm (w) × 6 mm (l) × 15 mm (h)) was printed and then immersed in PBS for a set time period at 37 °C. The prepared hydrogels were carefully sealed in a 50 mL conical tube, then immersed in liquid N_2_ for 10 min, and frozen. After unsealing, lyophilization was performed at −80 °C for 3 d. All samples were cut with a sharp knife to provide a clear view of the interior. Samples were then secured on a stub using carbon tapes, and coated with platinum. The cross-sectional morphologies were imaged with an SU-8010 scanning electron microscope (Hitachi; Tokyo, Japan). The pore size was measured by analyzing all lyophilized pores in the SEM image at 100× magnification.

### 2.9. Mechanical Testing

To confirm the structural stability of the Z-stacked GelMA scaffolds over the course of time—1, 3, and 5 d—cylinder model scaffolds (8 mm (d) × 2 mm (h)) were printed according to the procedure described in Section 2.4. A CT3 Texture analyzer (Brookfield; Toronto, ON, Canada) with a 4500 g load cell (Brookfield; Toronto, ON, Canada) in compression mode was used to measure the compressive strength of scaffolds. A probe 12.7 mm in diameter was used to compress with a trigger load of 0.05 N at a test speed of 0.05 mm/s. The compressive modulus was determined as the slope of the linear region corresponding to 5~15% strain.

### 2.10. Cell Adhesion

For cell adhesion studies, cylinder model scaffolds (8 mm (d) × 2 mm (h)) were printed according to the procedure described in Section 2.4. The medium was changed every day for 5 d. After 5 d, the cells were fixed and stained with rhodamine-phalloidin (Invitrogen, CA, USA) and counter-stained with 4’,6-diamidino-2-phenylindole (DAPI) to visualize F-actin filaments and cell nuclei, respectively. Green fluorescent protein (GFP) and DAPI were visualized using a fluorescence microscopy imaging system (Lionheart FX; BioTek Instruments, Winooski, VT, USA) equipped with a GFP and DAPI filter cube. The images were edited and organized into a montage of all z-stack images acquired at multiple focal points at 4× magnification.

### 2.11. Live/Dead Fluorescence Assay

To investigate the viability of the cells in the Z-stacked scaffold, the samples were prepared using the method described in Section 2.4. Cell viability was investigated using calcein AM/ethidium homodimer Live/Dead assay kits (Invitrogen, Carlsbad, CA, USA). After 1, 3, and 5 d of incubation, 1 mL of the staining solution was added according to the manufacturer’s protocol, and cells were imaged using a Lionheart FX microscope. The imaged cells were counted using Gen5 software (supplied with the Lionheart FX; BioTek Instruments, Winooski, VT, USA). The ratio of live cells to the total number of cells was used as a metric of cell viability.

### 2.12. Cell Proliferation Assay

To further investigate the cell proliferation and cytotoxicity of the Z-stacked scaffold, cell viability was investigated using Cell Counting Kit-8 (CCK-8; Dojindo, Kumamoto, Japan) and water-soluble tetrazolium (WST-1; Dojindo, Kumamoto, Japan). The samples were prepared as described in Section 2.4. Two samples were cultured without any change in media. After 1, 3, and 5 d of incubation, the CCK-8 and WST-1 assays were performed according to the manufacturer’s protocol. After incubation at 37 °C for 2 h, the absorbance of the samples was measured at 450 nm using a microplate spectrophotometer (BioTek Instruments, Winooski, VT, USA).

### 2.13. Statistical Analysis

To evaluate statistical significance, ordinary one- and two-way analyses of variance (ANOVAs) followed by Tukey’s test were performed. Data are presented as the mean ± standard deviation (SD), and means were compared using unpaired Student’s t-tests. A *p*-value of less than 0.05 was considered to indicate statistical significance. All analyses were performed using GraphPad Prism 8.0.2 (GraphPad Software; La Jolla, CA, USA).

## 3. Results and Discussion

### 3.1. ^1^H Nuclear Magnetic Resonance Spectroscopy

Methacrylation of gelatin was further verified using ^1^H NMR. Compared with the spectrum of unmodified gelatin, the GelMA sample showed new functional groups, marked as red (a) and green (c) in Figure 1A,B. The peaks at around 5.3 and 5.5 ppm chemical shifts were assigned to the acrylic protons of the grafted methacryloyl group, and the peak at 1.9 ppm was attributed to the methyl group of the grafted methacryloyl group. There was a decrease of intensity of the peak 2.9~3.1 ppm, which was assigned to lysine methylene (marked as blue (b)). As lysine is the target site for the reaction, this result was used to quantify the degree of methacrylation, which was estimated to be 81.4%.

### 3.2. Z-Stacking Strategy for Complex GelMA Scaffolds

**Figure 2:**
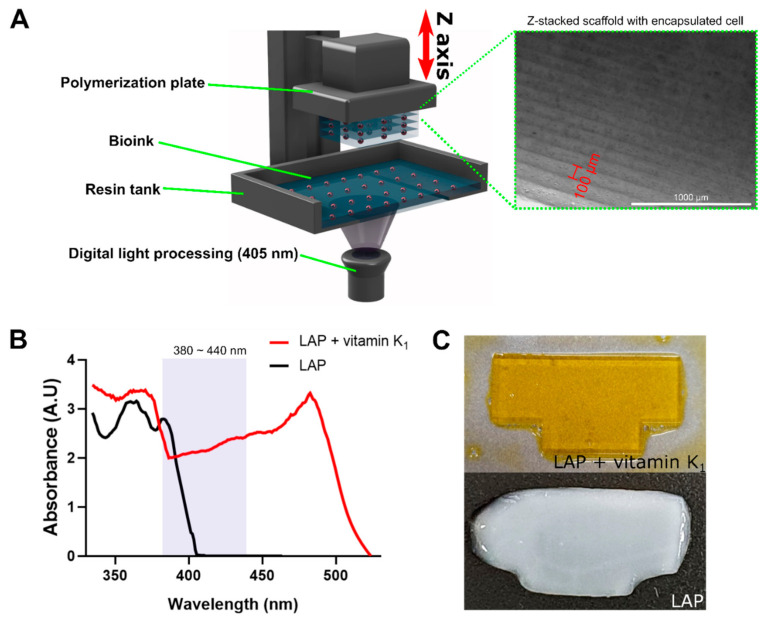
(**A**) Schematic describing the Z-stack bioprinting process for fabricating structures using bioink. LED light illuminates the resin tank from below, causing the resin to build up in units of 100 μm under the polymerization plate while inducing photo-crosslinking. (**B**) Absorbance spectra of only photoinitiator (LAP) and a mixture of photoinitiator and photo-absorber (LAP + vitamin K_1_). The shaded rectangular area is the LED wavelength range of the Z-stack bioprinter (380~440 nm). (**C**) Printing results using a combination of photoinitiator and photoabsorber (yellow, top) and only photoinitiator (white, bottom).

Figure 2A shows a schematic illustration of the fabrication of Z-stacked scaffolds using bioink. During the printing process, the resin tank was preheated and maintained at 37 °C to preserve cell viability. After transferring the low viscosity bioink (GelMA + BEFCs) into the resin tank, the polymerization plate was immersed in the resin tank, and the bioink was fabricated on the polymerization plate using DLP technology. Each layer was printed as slices of 100 μm thickness (Figure 2A). However, there was a mismatch between the response wavelength band of the LAP used as the photoinitiator and the wavelength band of the bioprinter output LED. The bioprinter mainly outputs an LED wavelength band between 380 and 440 nm, but the LAP does not react to radiation in this wavelength range. To solve this problem, vitamin K1 was used as a photoabsorber. A photoabsorber is used to control the penetration depth of incident light by adjusting the reaction wavelength band [20,21]. In this study, vitamin K_1_ was used as photoabsorber to adjust the photo-reaction wavelength band of the bioink, such that it responded to wavelengths in the range of 380 nm to 440 nm (Figure 2B). There was a clear difference between the results of printing with bioink prepared using only LAP as a photoinitiator and those obtained using bioink mixed with the photoabsorber. When only LAP was used, photo-crosslinking was either insufficient or excessive, resulting in poorly resolved printing. When the photoabsorber was used along with LAP, photo-crosslinking polymerization was stable, and the prints showed well-resolved features with fine edges. The addition of a photoabsorber is therefore important to prevent unwanted over-polymerization of the bioink (Figure 2C).

### 3.3. Printing Accuracy Analysis

**Figure 3:**
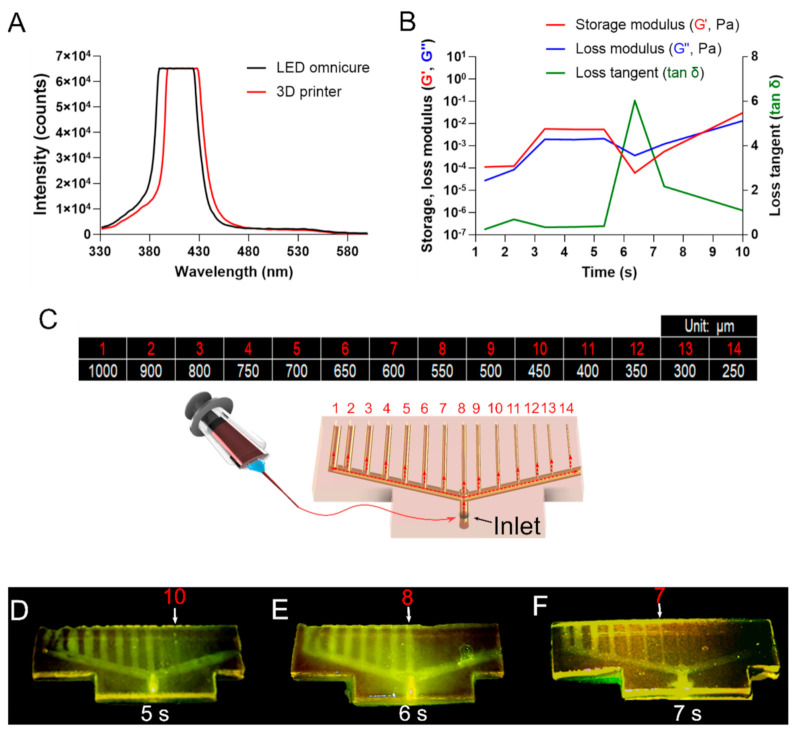
(**A**) Comparison of wavelength bands of output light by Z-stack bioprinter and LED OmniCure. (**B**) Photo-rheological property of the bioink. (**C**) Schematic showing the printing accuracy analysis model of hollow tubes with 14 different diameters. The table shows the diameter of each hollow tube. (**D**–**F**) Images of the scaffold printed models at different light exposure times: 5, 6, and 7 s.

Owing to the photopolymerizable characteristics of bioink, the printing accuracy differs according to the light exposure time [22,23]. To optimize the printing accuracy, photorheological analysis was performed to investigate the gelation kinetics of bioink. To conduct the experiment under conditions similar to the GelMA 3D printing environment, an LED OmniCure with an emission wavelength similar to that of the Z-stack bioprinter was prepared (Figure 3A). Photorheological analysis of the bioink showed that it had a tendency to increase and then maintain storage modulus (G’) and loss modulus (G’’) at the beginning of exposure to light (0–5.2 s), and there was no change in the loss tangent (tan δ). However, G’ decreased faster than G’’, and tan δ showed a sharply rising curve at 5.3 s. At 6.4 s, G’ rapidly rebounded, and tan δ decreased. Therefore, it was confirmed that the bioink was able to initiate the polymerization reaction from 5.3 s after the light exposure and up to 6.4 s (Figure 3B). To confirm whether the photorheology results were applicable in the Z-stack printing process, a model with 14 different hollow tubes was designed and fabricated by Z-stacking. The hollow tube model was adopted, as it is difficult to reproduce without the optimized crosslinking conditions because of the characteristics of the Z-stack bioprinter (Figure 3C). The hollow tube models were printed and observed under three light exposure time periods: 5, 6, and 7 s. The scaffold fabricated under 5 s light exposure could be identified from 1000 μm (tube 1) to 450 μm (tube 10) in diameter. Although it was possible to implement even a fine tube of 450 μm, structural collapse occurred due to insufficient polymerization (Figure 3D). The sample with 6 s exposure displayed a structurally stable scaffold, and the modeling reproducibility was high in this case. Hollow tubes were available from 1000 μm (tube 1) to 550 μm (tube 8) in diameter, and no excessive polymerization was observed (Figure 3E). Under 7 s of light exposure, the scaffold was structurally stable. However, most of the hollow tubes were blocked due to over-polymerization (Figure 3F). No significant difference was found from the results obtained through photorheology analysis. The optimal light exposure time was therefore optimized to produce complex structures, similar to those of the native tissue, with high reproducibility.

### 3.4. Scanning Electron Microscopy (SEM)

**Figure 4:**
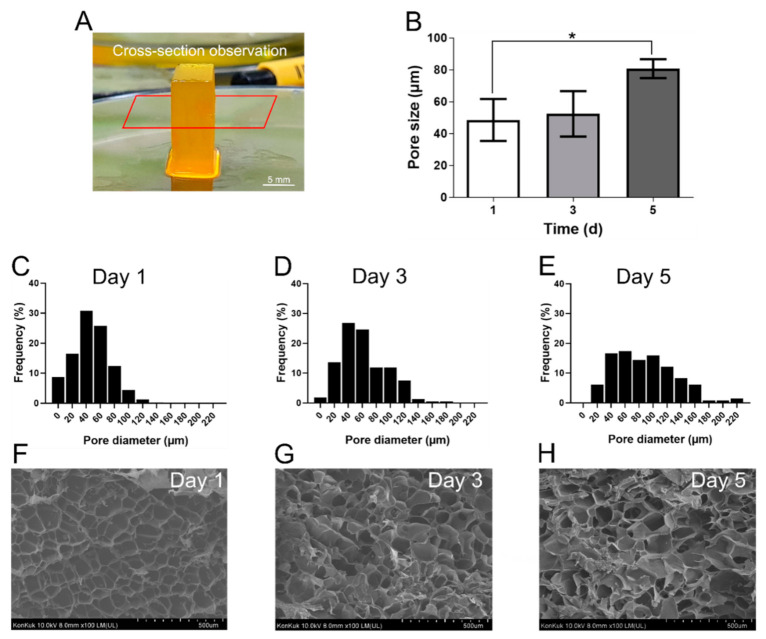
(**A**) Test sample for cross-section and observation planes (red rectangle). (**B**) Average pore size according to time courses: 1, 3, and 5 d. * *p* < 0.05 between the indicated groups. Data are shown as means ± SD, *n* = 3. (**C**–**E**) Pore distribution frequency obtained from SEM images of Z-stacked scaffold prepared under different time points: 1, 3, and 5 d. (**F**–**H**) SEM images of pore structure according to different time points: 1, 3, and 5 d. The magnifications of SEM images are ×100.

The pore structure within a hydrogel has been shown to be important for providing a cell niche and controlling cell differentiation [24,25]. Porosity and interconnected pore networks are essential for supporting nutrient diffusion via convection in cell culture media within the fabricated scaffold [26]. The pore structure of the printed scaffold should also provide a cell niche and include additional space for cell proliferation. Therefore, the morphological characteristics of the cellular microenvironment in the printed scaffold were investigated using SEM. As shown in Figure 4A, the cross-sectional microstructures of the printed scaffold were imaged using SEM. The average pore size of the Z-stacked scaffold in the day 1 group was 48.5 ± 13.1 μm. The average pore size had slightly increased by day 3 (52.4 ± 14.2 μm). A statistically significant difference in pore size was observed in samples from day 5 (80.7 ± 5.9 μm) as compared to those from day 1 (Figure 4B). The measured pore size does not represent the actual pore size of the hydrogel, but it allows for a relative comparison and indirect prediction of the pore size. Over the time course, it was not possible to maintain a regular shape, due to structural collapse, and the pore distribution tended to increase due to the increase in large pores. On day 1, the pore network retained a certain size range and a consistent porous structure (Figure 4C,F). On day 3, pore expansion occurred due to swelling, but the overall pore size distribution was similar (Figure 4D,G). On day 5, the number of large pores increased greatly, as structural collapse occurred because of the biodegradation and swelling of GelMA and the large amount of swelling that occurred at physiological temperature [13,27,28]. Spatial expansion was therefore confirmed (Figure 4E,H). Based on these results, we confirmed the inner pore structure of the Z-stacked scaffold provided space for cell proliferation over time.

### 3.5. Mechanical Properties

**Figure 5:**
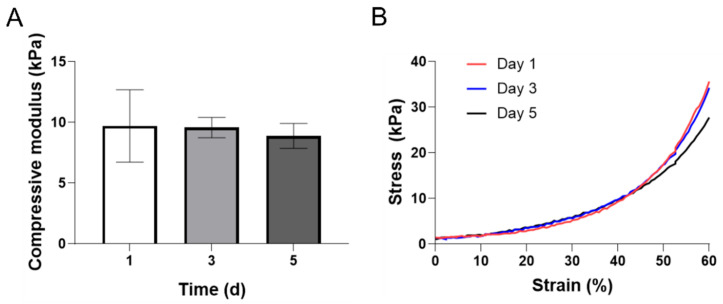
(**A**) Compressive modulus of Z-stacked GelMA scaffold according to time courses: 1, 3, and 5 d. There were no statistically significant differences. Data shown are the mean ± SD, *n* = 5. (**B**) A representative stress-strain curve of Z-stacked GelMA scaffold according to time courses: 1, 3, and 5 d.

The compressive modulus was measured to determine the effect of the increase in pore size over time on the structural stability of the scaffold. The compressive modulus of the scaffold sample of the day 1 group was 9.7 ± 2.9 kPa. Day 3 and 5 groups showed no statistically significant difference, 9.5 ± 0.8 kPa and 8.9 ± 1.0 kPa, respectively. The compressive modulus tended to decrease with the increase of the pore diameter with time. However, the compressive modulus did not decrease as much as the pore diameter increased (Figure 5A). This decreasing trend was also observed in the strain-stress curve. There was no difference between groups in the initial strain (~45%). In strain later point (45~60%), there was a difference in the stress curve. Day 1 group showed the fastest increase, and day 5 group showed the slowest increase trend. However, there was no significant difference in this curve (Figure 5B). As a result, the increase in pore size did not have a significant effect on the mechanical properties.

### 3.6. Cell Adhesion in Z-Stacked Scaffolds

**Figure 6:**
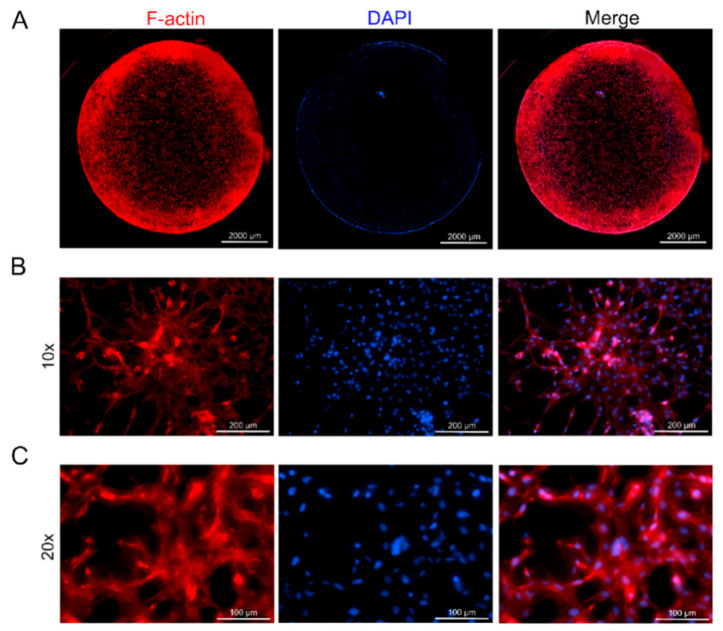
Phalloidin labeled F-actin (red), DAPI nuclear staining (blue) and overlaid fluorescent image of Z-stacked scaffold (merged) for BEFCs on day 5. (**A**) Full image of the scaffold sample. Scale bar = 2000 µm. (**B**) 10× magnification of scaffold sample. Scale bar = 200 µm. (**C**) 20× magnification of scaffold sample. Scale bar = 100 µm.

Cellular adhesion within 3D scaffolds is essential for the long-term culture of encapsulated cells in engineered tissues [4]. To confirm cell adhesion and cell-to-cell network formation in the multi-layered Z-stacked scaffold, the morphology of the encapsulated cells was observed using F-actin and DAPI staining. As can be seen in the images of BEFCs cultured in a scaffold for 5 d, encapsulated BEFCs actively formed microfilaments (Figure 6A). We also confirmed that the cells were evenly distributed throughout the scaffold. In addition, in the 10× and 20× images, it was confirmed that the cells adhered within the 3D space in all directions (Figure 6B,C). Thus, it was confirmed that the Z-stacked scaffold can facilitate 3D cell adhesion.

### 3.7. Cell Viability

**Figure 7:**
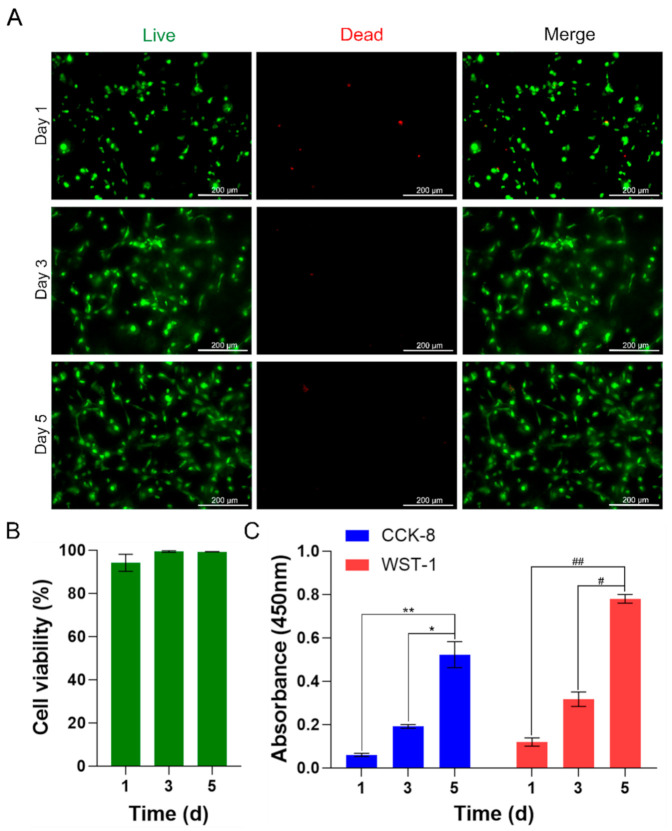
(**A**) Fluorescence microscopy images of Live/Dead assay. BEFCs cultured in Z-stacked scaffolds after 1, 3, and 5 d. Green indicates live cells and red indicates dead cells. The merged images indicate live and dead cells together. Scale bar = 200 µm. (**B**) Cell viability obtained from Live/Dead assay of Z-stacked scaffold at different time points: 1, 3, and 5 d. There were no statistically significant differences. Data shown are the mean ± SD, *n* = 4. (**C**) CCK-8 and WST-1 absorbance graph of Z-stacked scaffold on days 1, 3, and 5. * *p* < 0.05, ** *p* < 0.01; # *p* < 0.05, ## *p* < 0.01 between the indicated groups. Data shown are the mean ± SD, *n* = 8.

The viability and morphology of encapsulated BEFCs were investigated for 5 d to determine whether the Z-stacked scaffolds were suitable for tissue engineering applications. The test was conducted to confirm whether the cells were supplied with sufficient nutrients and oxygen to maintain cell viability in the Z-stacked scaffolds when the scaffolds contained a high concentration ($2\times 10^{6}$ cells/mL) of cells (Figure 7A). On day 1, the calculated cell viability was high, at 94.2 ± 3.9%, indicating that stable encapsulation of cells was achieved during the bioink fabrication and printing process. After 3 d, the cell viability reached 99.5 ± 0.4%, and more cells were observed as a result of the proliferation of existing cells. Interconnected networks with neighboring cells were also seen. After 5 d of incubation, small multicellular networks and elongated cells were observed within the 3D space. The cell viability of BEFCs in the Z-stacked scaffold on day 5 was 99.3 ± 0.07% (Figure 7B). The Z-stacked scaffold maintained excellent cell viability for at least 5 d, by providing the cells with a favorable 3D cellular microenvironment.

### 3.8. Cell Proliferation Analysis

The proliferation of BEFCs encapsulated in Z-stacked scaffolds was investigated for 5 d using both CCK-8 and WST-1 assays. On days 1, 3, and 5, BEFCs encapsulated in Z-stacked scaffolds proliferated effectively. CCK-8 assay revealed an increase in cell counts by 220.04% on day 3 and 774.76% on day 5, compared to day 1. Results of WST-1 assays showed an increase by 164.98% on day 3 and 554.41% on day 5, compared to day 1. The results of the CCK-8 and WST-1 assays demonstrated that the Z-stacked scaffold provided a favorable environment for cell encapsulation and proliferation (Figure 7C).

### 3.9. Ear-Shaped Z-Stacked Scaffold Printing and Cell Culture

**Figure 8:**
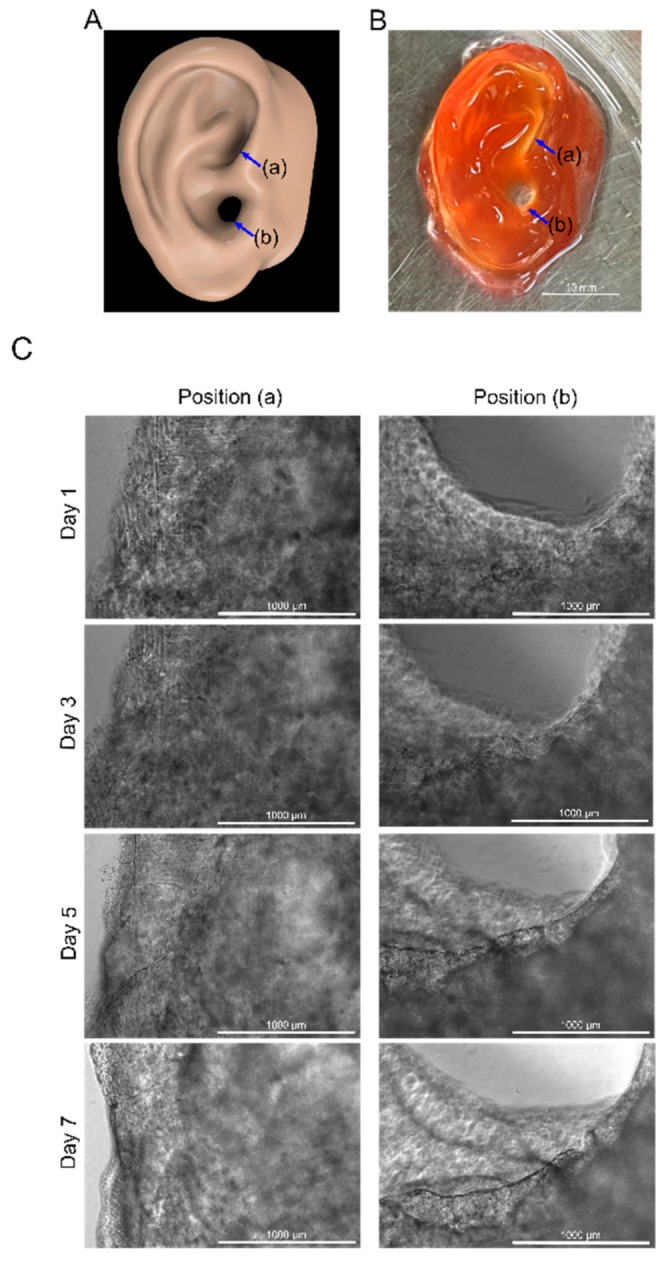
(**A**) 3D ear model. (**B**) Ear-shaped Z-stacked scaffold. Scale bar = 10 mm. (**C**) Phase contrast 4x magnification microscope image of position (a) and (b) of the scaffold from 1 to 7 d. Scale bar = 1000 µm.

A human ear design was printed using the bioink loaded with BEFCs to reproduce the intricate structure of the tissue and confirm the value of the Z-stacked scaffold (Figure 8A). In general, loss in cell viability due to nutrient depletion and drying are encountered during the fabrication of large scaffolds because of the extended 3D printed process. The optimized bioink and printing parameters obtained using the present method not only enabled higher resolution and model reproducibility but also allowed rapid printing, preventing nutrient restriction and drying while printing large build sizes. The resulting ear-shaped Z-stacked scaffold showed a smooth surface, and the complex curvature structures such as the ear hole and pinna were reproduced with a high degree of accuracy (Figure 8B).

Cells encapsulated in the scaffold were observed, starting on day 1 of culture. Individual elongated cells were evenly distributed in the 3D space, and started to form interconnected networks with neighboring cells. On days 3 and 5 of the culture, the cells distributed in each layer proliferated extensively. By day 7, active cell proliferation and migration resulted in the formation of a dense cellular network (Figure 8C; Supplementary Video S1–8). Thus, a macroscale, cellular Z-stacked scaffold was created using the optimized conditions. The input model could be accurately reproduced by adjusting the light exposure time and the wavelength band of the bioink. Long-term cell proliferation and the formation of a multicellular network were apparent.

## 4. Conclusions

In this study, GelMA-based bioinks were developed and optimized for artificial tissue engineering applications using a Z-stack bioprinter to achieve excellent model reproducibility and biocompatibility. We optimized the reactivity of the light source and the extent of polymerization of the Z-stack bioprinter by adding a photoabsorber (vitamin K_1_), thereby improving the printing accuracy through light exposure time optimization. Z-stacked GelMA scaffolds fabricated in an optimized environment were found to have an internal porous structure suitable for the encapsulation of cells. This internal structure facilitated formation of additional space over time, but it did not threaten the structural stability of the scaffold. GelMA scaffolds facilitated cell adhesion and proliferation, while ensuring high cell viability during the entire culture period. Based on these data, an ear-shaped Z-stacked scaffold with a real scale and complex morphology was fabricated. The results showed that the native tissue structure was fully embodied and can be fabricated while ensuring cell survival and proliferation for seven days. From the results of high-speed printing with cells using GelMA as the sole polymer, the highly biocompatible and highly reproducible Z-stacked scaffold can be considered a manufacturing technology with high potential for various applications in tissue engineering.

## Supplementary Materials

The following are available online at https://www.mdpi.com/2073-4360/12/12/3027/s1, Videos S1–8: 3D model of long-term cultured ear-shaped Z-stacked scaffold.
